# Lecithin is the key material attribute in soy bean oil affecting filamentous bioprocesses

**DOI:** 10.1186/s13568-018-0625-0

**Published:** 2018-06-01

**Authors:** Alexandra Hofer, Christoph Herwig, Oliver Spadiut

**Affiliations:** 1 Research Area Biochemical Engineering, Institute of Chemical, Environmental and Bioscience Engineering, Vienna University of Technology, Gumpendorferstrasse 1a - 166/4, 1060 Vienna, Austria; 2 CD Laboratory on Mechanistic and Physiological Methods for Improved Bioprocesses, Vienna University of Technology, Gumpendorferstrasse 1a - 166/4, 1060 Vienna, Austria

**Keywords:** Complex raw material, Key material attribute, Soy bean oil, Lecithin, Filamentous fungi

## Abstract

Complex raw materials are widely used as supplements in biopharmaceutical production processes due to their positive effect on biomass growth and productivity at low cost. However, their use negatively impacts process reproducibility due to high lot-to-lot variability which contradicts current regulatory guidelines. In this study we investigated crude soy bean oil (SBO) which is a common complex raw material for filamentous fungi. We demonstrated that lecithin, which we define as phosphatidylcholines, is in fact the key material attribute in crude SBO positively affecting fungal growth and consequently productivity. The methodological toolbox we present here allows the straightforward isolation of lecithin from crude SBO, its semi-quantification by HPLC and the consequent supplementation thereof in defined amounts. Thus, over-dosage and potential resulting negative impacts on fungal growth and productivity can be omitted.

## Introduction

Complex raw materials are commonly used as cheap media supplements in bacterial, fungal and mammalian bioprocesses (Gao and Yuan 2011; Millis et al. 1963; Reese and Maguire 1969). These raw materials are typically of biological origin and thus underlie a high lot-to-lot variability. Furthermore, the specific substance responsible for the positive impact on the bioprocess is often unknown. However, during the International Conference of Harmonization, guidelines have been established demanding for the evaluation of critical material attributes (CMAs) and their impact on product quality (ICH 2009). Material attributes do not only affect product quality but also process performance and productivity. We define these material attributes that have an impact on productivity as key material attributes (kMAs). For both, identification of CMAs and kMAs, sound science-based knowledge of raw material quality has become a necessity.

An example for complex biological raw materials are vegetable oils, such as soy bean oil (SBO). It was shown that SBO leads to an increase in fungal biomass growth and a 50% increase in antibiotics production (Goldschmidt and Koffler 1950; Pan et al. 1959). Although the reasons for this positive effect are still obscure, possible explanations are: (i) SBO serves as nutritional source, (ii) SBO changes the medium for improved oxygen transfer, and (iii) the antifoam capacity of the oil reduces microbial cell damage (Goldschmidt and Koffler 1950; Jones and Porter 1998). Two critical factors for SBO supplementation are known, namely (i) the time point of addition and (ii) the dosage (Anderson et al. 1956; Ohta et al. 1995). It has been shown that low-level supplementation of SBO is beneficial, whereas medium to high-level supplementation is disadvantageous. To shed more light on that, the effect of crude and refined SBO was tested, however no significant differences in the production of tetracycline were found (Jones and Porter 1998). Although crude SBO is about 1/5 cheaper than refined oil and contains phospholipids, some minerals and tocopherols, it may also contain pesticides, which is why refined SBO is mostly used as medium supplement today. However, several studies have shown the positive impact of phospholipids in crude SBO on fungal growth and productivity (Bateman and Jenkins 1997; Goldschmidt and Koffler 1950; Tarafdar and Claassen 1988). We hypothesize that lecithin, which we define as phosphatidylcholines, is the kMA in SBO causing this positive impact. To investigate this hypothesis, we isolated lecithin from SBO, added defined amounts thereof to the media for a *Penicillium chrysogenum* strain and analyzed the effects on fungal growth. For the extraction of lecithin from oily matrices several solid phase extraction (SPE) methods have been reported (Bateman and Jenkins 1997; Bligh and Dyer 1959; Carelli et al. 1997; Nash and Frankel 1986; Ruiz-Gutierrez and Perez-Camino 2000). However, SPE is quite tedious and time consuming and delivers the wanted lecithin in an organic solvent, which is commonly not suitable for media supplementation. Hence, we adopted a simple and cheap extraction procedure, which is currently applied in industrial refinery processes (Dijkstra and Van Opstal 1989), delivering lecithin in an aqueous background. Additionally, we present a semi-quantitative HPLC-CAD method for crude SBO and lecithin analysis, which allows dosage of the extracted lecithin without the need of mass spectrometry analysis. The effect of crude SBO and extracted lecithin on fungal growth was evaluated in shake flask experiments with *P. chrysogenum*. Summarizing, in this study we demonstrate that lecithin is in fact the kMA in SBO affecting fungal growth and consequently productivity.

## Materials and methods

### Chemicals

All chemicals were of analytical grade and purchased from Carl Roth (Karlsruhe, Germany) or Sigma Aldrich (St. Louis, MO, USA). Ultra-pure water derived from a Milli-Q system from Merck Millipore (Billerica, MA, USA) was used for analytical methods.

### SBO samples

Unrefined soy bean oil (SBO) originated from two European manufacturers (called M1 and M2).

### SBO lot-to-lot variability

For demonstration of SBO lot-to-lot variability, 20 samples from manufacturer M1 and M2 were analyzed for free fatty acid (FFA), fatty acid methyl esters (FAME) and phosphorous content as well as for a spectroscopic fingerprint by Fourier transform infrared spectroscopy (FTIR). Data were analyzed by principal component analysis (PCA).

Furthermore, our industrial cooperation partner delivered 29 SBO samples as well as on-line and off-line data of an industrial scale fungal production process. In order to assess the impact of SBO variability on process performance, multivariate data analysis was performed, assessing correlations between SBO composition and byproduct as well as product formation.

### Analytical methods

#### HPLC method for crude SBO and lecithin

Chromatographic separation was accomplished by adopting the method from Jangle et al. (2013) using a Zorbax Eclipse Plus C-18 column (3.0 × 150 mm, 3.5 µm; Agilent Technologies) and pre-column (4.6 × 12.5 mm, 5 µm). Further details of the chromatographic method are given in the Results section. An Ultimate 3000 HPLC system (Thermo Fisher Scientific, Waltham, MA, USA) equipped with a pump (LPG-3400SD), an autosampler (WPS-3000 SplitLoop), a column oven (Col.Comp. TCC-3000SD), a diode array detector (DAD 3000) and a charged aerosol detector (Corona Veo RS) was used. Chromeleon 7.2 was used for control.

#### HPLC method for free fatty acids (FFA)

For sample preparation, FFA were extracted with butyl acetate at room temperature. The separation of FFA, namely 18:0, 18:1, 18:2 and 18:3, was achieved by a Zorbax Eclipse Plus C-18 column (3.0 × 150 mm, 3.5 µm; Agilent Technologies) and pre-column (4.6 × 12.5 mm, 5 µm). A gradient was implemented over 4 min, starting from 90% MQ to 100% acetonitrile, which was held for another 6 min. FFA were detected by a charged aerosol detector. The method parameters were: 1.0 ml/min flowrate, 50 °C column oven temperature and 2 µl injection volume.

#### GC method for fatty acid methyl esters (FAME)

An Agilent Gas Chromatography system (7890A, Agilent Technologies, Santa Clara, California, USA) with a flame ionisation detector (FID) was used. Chromatographic separation was achieved by an HP-88 column (60 m × 0.25 mm ID, 0.2 µm; Agilent) and a temperature gradient starting at 40 °C up to 220 °C within 80 min. 1 µl was injected with an inlet split of 10:1. Prior to injection, the samples were derivatised with trimethylsulfonium hydroxide (TMSH) for esterification. Therefore 200 µl sample, 200 µl internal standard solution and 200 µl TMSH were incubated at 50 °C for 15 min. Quantification was achieved via external and internal standards, e.g. 19:0. Standards were prepared of 14:0, 16:0, 16:1, 18:0, 18:1, 18:2 and 18:3 fatty acids.

#### ICP-OES for phosphor analysis

The samples were diluted in PremiSolv^®^ (SPrep, Germany) and indium was added as internal standard. Samples were analyzed on an iCAP 6000 inductively coupled plasma—optical emission spectrometer (ICP-OES, Thermo Scientific). The instrument was equipped with a radial optic, an Echelle spectrometer and a CCD-chip detector. The samples were brought into the plasma using a Babington-type nebulizer and a peristaltic pump.

#### FTIR analysis for SBO fingerprint

Crude SBO samples were directly analyzed using an FTIR instrument equipped with an ATR Platinum, a Tensor 37 photometer and a DTGS detector (Bruker optics). Spectra were acquired with a resolution of 4 cm^−1^ and 32 scans per sample against a background of air. The acquired wavenumber region ranged from 600 to 4000 cm^−1^.

### Shake flask cultivations

A P-14 *P. chrysogenum* candidate strain, descending from the P-2 *P. chrysogenum* candidate strain (American Type Culture Collection with the access number ATCC 48271), was used for all experiments. The cultivation medium was similar as described previously (Posch et al. 2013), either with or without supplementation of crude SBO or extracted lecithin. In every case glucose was the main C-source. For preculture, 500 ml shake flasks were filled with 30 ml medium and inoculated with 2.8 × 10^8^ spores of *P. chrysogenum*. The flasks were incubated at 25 °C and 300 rpm for up to 72 h. A flask filled with destilled water was added in the incubator in order to increase humidity and reduce evaporation. The flasks were either harvested at the end-point only after 72 h or more sample points were evaluated in order to get time-resolved data. For the latter, samples were analyzed every 12 h. Due to the low volume in the flasks, for every time point two separate shake flask were analyzed.

If a main culture for product formation was added after the preculture, the preculture was transferred already after 55 h. 3 ml of preculture were transferred into 27 ml of main culture medium and incubated at 25 °C and 300 rpm for 120 h.

For determination of cell dry weight (CDW) 5 ml of broth were pipetted to preweighed glass tubes, which were centrifuged at 4800 rpm and 4 °C for 10 min. After decantation, the pellet was washed with 5 ml distilled water and centrifuged again. The supernatant was discarded and the pellet was dried at 95 °C for 72 h. The CDW was then analyzed gravimetrically. CDW analysis was performed in triplicates. Product titer determination of the main culture supernatant was performed by HPLC according to (Posch et al. 2012).

### Statistical analysis

The lot-to-lot variability of SBO samples was analyzed by principal component analysis (PCA) and partial least squares (PLS) regression using Umetrics SIMCA 4.0 (Umeå, Sweden). Before analysis, the dataset including the specific composition of SBO as well as the dataset including the byproduct concentration were normalized and mean-centered. From the spectral data, *i.e*. the fingerprints, the second derivative was generated in order to assure baseline correction and the data were mean-centered. For PCA, the final matrix consisted of 12 observations and the FTIR region between 800 and 2000 cm^−1^. For PLS analysis the x-matrix consisted of 39 observations and 12 specific components, namely FFA, FAME and phosphor, and the y-vector of 39 observations and the maximal byproduct concentration.

The design of experiment (DoE) investigating the impact of crude SBO and extracted lecithin as well as interactions on process performance was designed with MODDE Umetrics (Umeå, Sweden), choosing a full factorial screening with 2 factors, namely crude SBO and extracted lecithin, and 3 levels, namely 0, 4 or 8 g/l, and resulted in 11 experiments including 3 center points.

## Results

### SBO as typical complex raw material

**Fig. 1:**
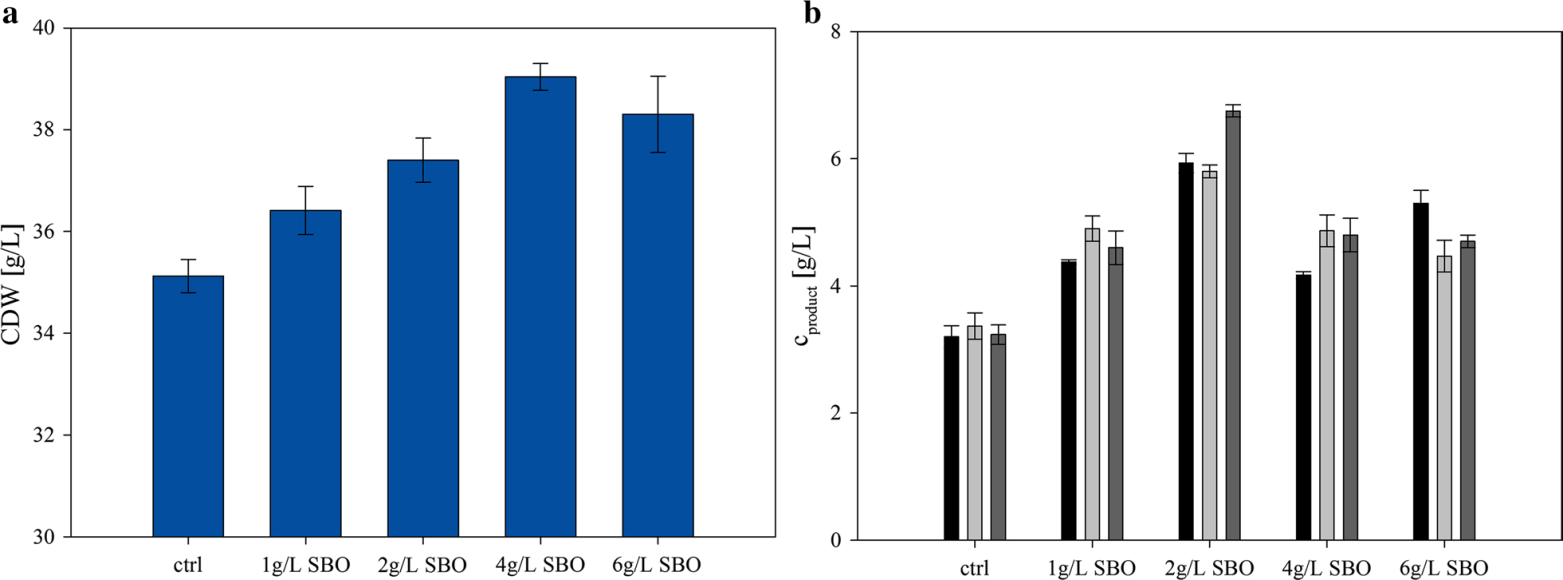
Two stage shake flask experiments with *P. chrysogenum* and crude SBO supplemented in the preculture media. At the transfer from preculture to main culture, 3 shake flasks were inoculated from one preculture medium in order to check reproducibility. Plot **a** shows the CDW after 55 h of preculture at the time point of transfer with respect to different amounts of added SBO and a control run without supplementation (ctrl). Plot **b** shows the corresponding product concentration at the end of the main culture

Typical characteristics of complex raw materials are their positive impact on growth and productivity at low cost, but also their negative impact on process reproducibility due to high lot-to-lot variability. We assessed both characteristics for crude SBO in a *P. chrysogenum* antibiotics production process in shake flask experiments (Fig. 1). In the preculture, supplementation with up to 4 g/l crude SBO led to an increase in biomass formation. A higher concentration of SBO had a negative effect. This dosage-dependent effect had been reported before, however reasons therefore are still unknown (Jones and Porter 1998). We speculate that a limited oxygen transfer due to the oil or too high concentrations of unprofitable ingredients in the crude oil might be the reason. In the main culture phase the supplementation with crude SBO led to an increase in product concentration up to almost 50%. The specific productivity increased by 25% for 1 and 4 g/l and by 45% for 2 g/l. Again, specific productivity increased the lowest when 6 g/l crude SBO were added, namely only by 20% compared to the unsupplemented control. Summarizing, we clearly see a concentration-dependent positive impact of crude SBO on fungal growth and productivity.

### SBO lot-to-lot variability

**Fig. 2:**
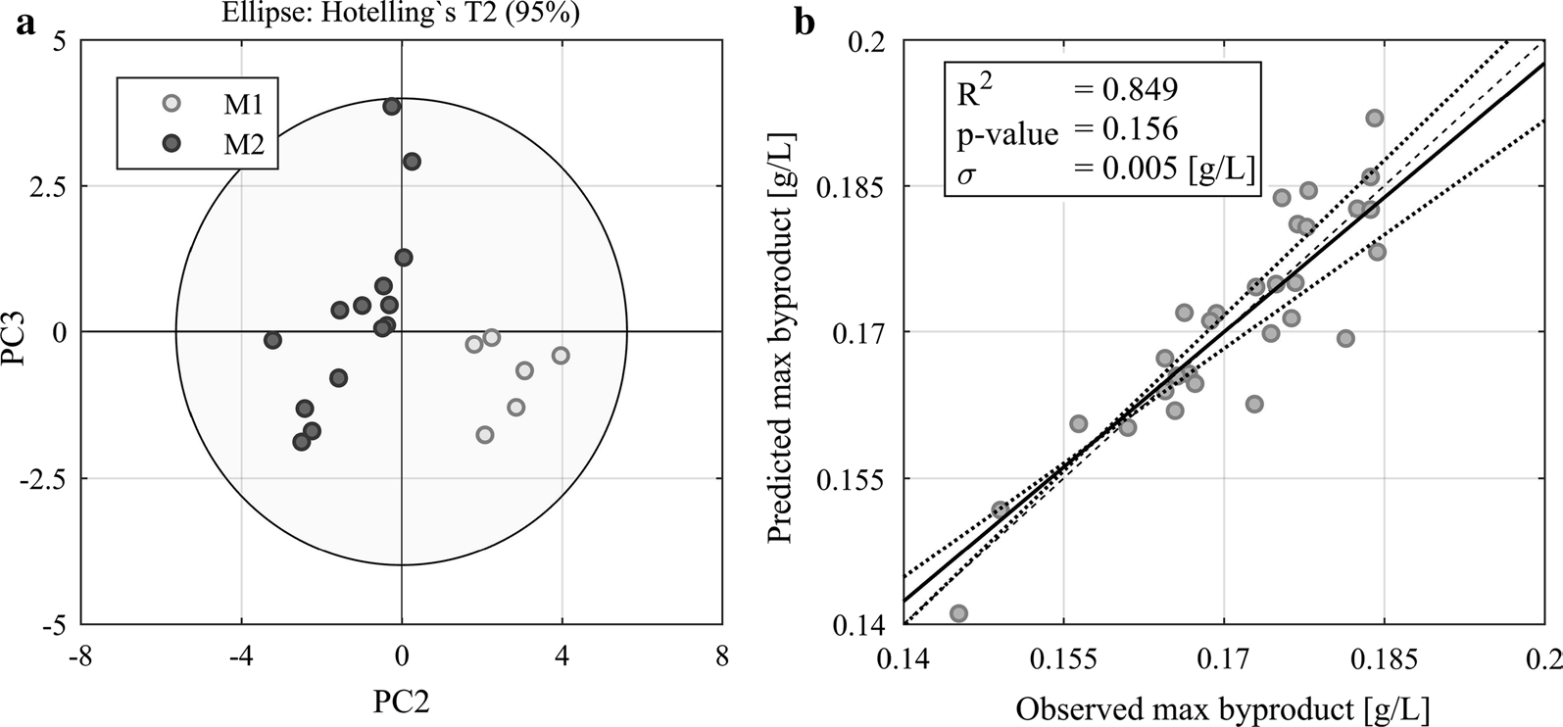
PCA of FTIR spectra of crude SBO samples from different manufacturers resulted in 4 PCs (R2 0.999, cum 1.000). The score plot shows clustering according to manufacturer along PC 2 (plot **a**). A PLS model of the specific composition of SBO and the byproduct resulted in 2 LVs (R2 0.29, 0.49) and showed some correlation between those variables. Hence, lot-to-lot variability of SBO seems to have an impact on process performance attributes (plot **b**). PCA of FTIR spectra of SBO samples from different manufacturers resulted in 4 PCs (R^2^ 0.999, cum 1.000). The score plot shows clustering according to manufacturer along PC 2 (plot **a**). A PLS model of the specific composition of SBO and the byproduct resulted in 2 LVs (R^2^ 0.29, 0.49) and showed some correlation between those variables. Hence, lot-to-lot variability of SBO seems to have an impact on process performance attributes (plot **b**)

The lot-to-lot variability of crude SBO and the associated negative impact on the process were evaluated with SBO samples which were collected during an industrial fungal production process over one year. PCA of the FTIR fingerprinting spectra clearly showed clustering according to the manufacturer along PC2 (Fig. 2a). Analysis of the production data and the specific SBO composition by PLS showed that there was a certain correlation between the SBO composition and the formation of unwanted byproducts (Fig. 2b) as well as maximum product titer (data not shown). Clearly, crude SBO is characterized by a high lot-to-lot variability which significantly impacts fungal bioprocesses.

### Extraction and analysis of lecithin from crude SBO

We hypothesized that lecithin, which we defined as phosphatidylcholines delivering fatty acids, phosphor and glycerol, might be the kMA in crude SBO triggering the positive effect on fungal growth and productivity. To test this hypothesis we developed an extraction procedure as well as a quantification method, allowing the supplementation of defined amounts of lecithin to fungal cultures.

Commonly published methods for lecithin extraction, such as SPE approaches, are quite cumbersome. Hence, we decided to adopt the quite simple method of lecithin degumming, which is a method currently used in industry for oil refinery (Dijkstra and Van Opstal 1989; Wiedermann 1981). 4 ml crude SBO and 1 ml of distilled water were heated up separately to 80 °C in closed glass eprouvettes for 1 h. Then, the preheated liquids were immediately united and vortexed for 1 min. Afterwards, the eprouvettes were centrifuged at 5000 rpm at 20 °C for 10 min. The lower aqueous phase contained the lecithin.

To quantify the extracted lecithin we adopted a RP-HPLC method using a C18 column from literature (Jangle et al. 2013). The elution gradient was optimized, resulting in a total runtime of 20 min (Table 1). The flowrate was set to 0.5 ml/min and the column oven was set to 50 °C. Ten µl of the sample were injected and analyzed at 205 nm as well as with a charged aerosol detector (CAD) using the following settings: an evaporator temperature of 50 °C, 5 Hz data collection rate, a filter of 3.6 and a power function of 1.90.

**Table 1:** Gradient program for quantification of extracted lecithin

t (min)	0	1	2	6	10	10	20
% A	95	95	0	0	0	0	0
% B	5	5	100	100	50	30	30
% C	0	0	0	0	50	70	70

Eluent A is MQ water, eluent B is acetonitrile and eluent C is isopropanol

**Fig. 3:**
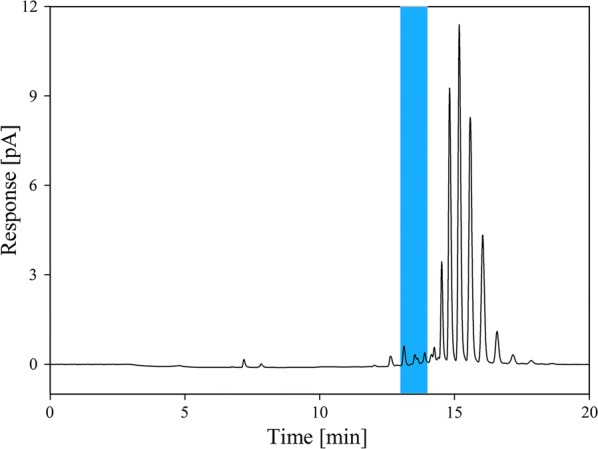
Chromatogram of crude SBO. The peak area at 13.6 min could be assigned to lecithin (marked in blue)

The method was assessed by injection of crude SBO and purchased lecithin standards. Lecithin was detected as various peaks at around 13 min (highlighted in Fig. 3). The reason for this is that lecithin is a mixture of different phospholipids, which show slightly different polarity. However, the method allows a relative quantification of extracted lecithin which was sufficient to test our hypothesis.

In order to evaluate the successful extraction and identification of lecithin from crude SBO, the extracted lecithin was dried and re-dissolved in MeOH for reference analytics. In short, HPLC chromatograms confirmed lecithin, which was also identified by ESI–MS (data not shown). Summarizing, simple degumming and HPLC quantification allow media supplementation with defined amounts of extracted lecithin to fungal bioprocesses.

### Effects of crude SBO versus extracted lecithin

**Fig. 4:**
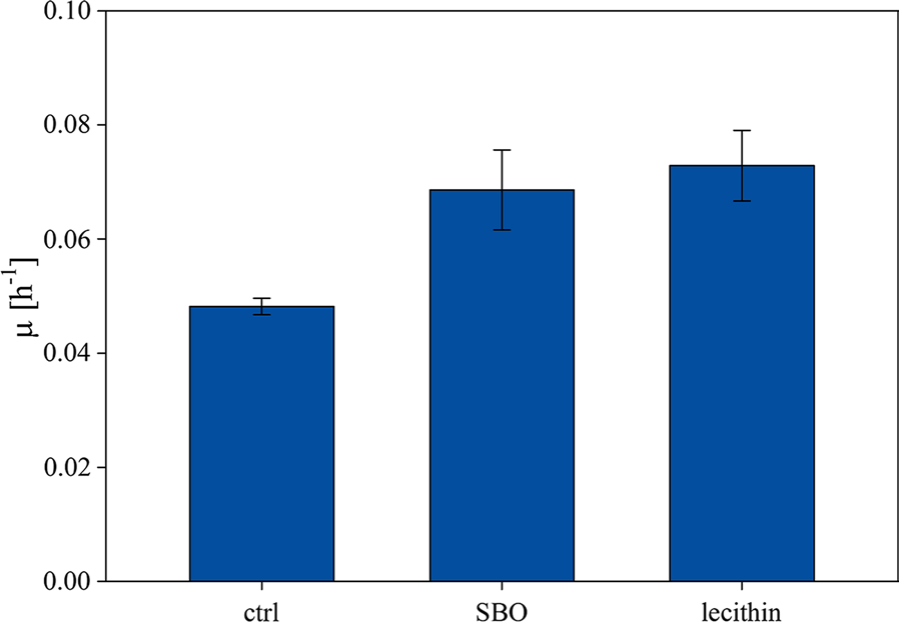
Shake flask experiments with a *P. chrysogenum* strain and different media supplementation. Media were either not supplemented or supplemented with crude SBO or an equivalent amount of extracted lecithin

In order to test our hypothesis that lecithin in crude SBO positively affects fungal bioprocesses, we performed several supplementation experiments in shake flasks and analyzed fungal growth. Time-resolved data for both supplements as well as for a control run without SBO or lecithin addition were analyzed over 72 h cultivation time. A positive effect of crude SBO as well as extracted lecithin on fungal cell growth was detected already after 24 h of cultivation. Both supplements increased biomass formation by 12–15% and a higher specific growth rate (µ) in comparison to the non-supplemented control was observed (Fig. 4). Notably, crude SBO and extracted lecithin showed comparable effects demonstrating that lecithin is in fact the kMA in crude SBO.

**Fig. 5:**
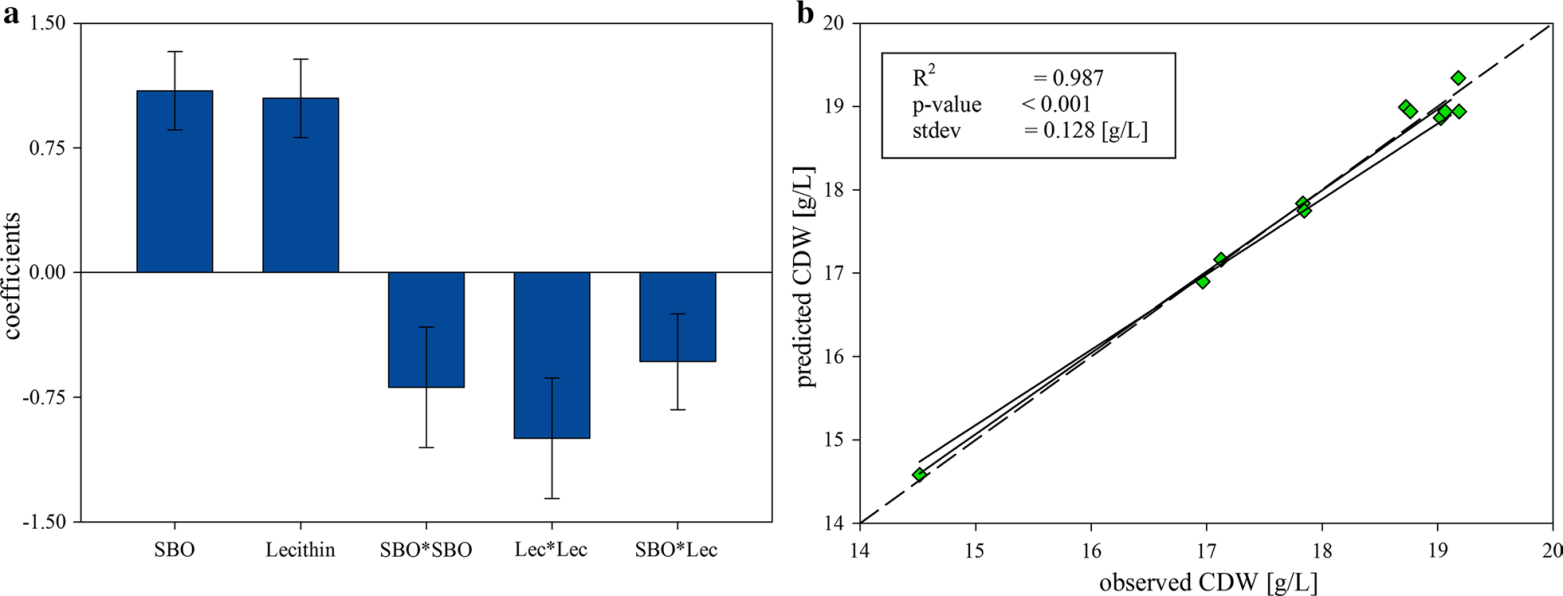
The data of the 23 DoE were fitted using an MLR model. The coefficient plot (plot **a**) shows that crude SBO as well as extracted lecithin positively correlate to the CDW. Nevertheless, too high concentrations seem to have a negative impact, which can be seen in the negative interaction and quadratic terms. Plot **b** represents the observed vs predicted plot of the model, showing a good model fit

In order to get more insights into the beneficial effects of crude SBO and extracted lecithin on biomass formation a DoE with 3 different concentrations of crude SBO and extracted lecithin, respectively, was performed (Fig. 5). For data analysis an MLR model was fitted including an interaction term and quadratic effects. Reliability of the model was demonstrated by a condition number of 3.082, R^2^ of 0.987, Q^2^ of 0.920, model validity of 0.827 and a reproducibility of 0.977. The results of the DoE substantiated former experiments with respect to the positive effect of both crude SBO and extracted lecithin on biomass formation and an upper limit of this positive effect when too much of the respective supplement is added to the medium (shown as negative correlation of interaction and quadratic terms; Fig. 5).

**Fig. 6:**
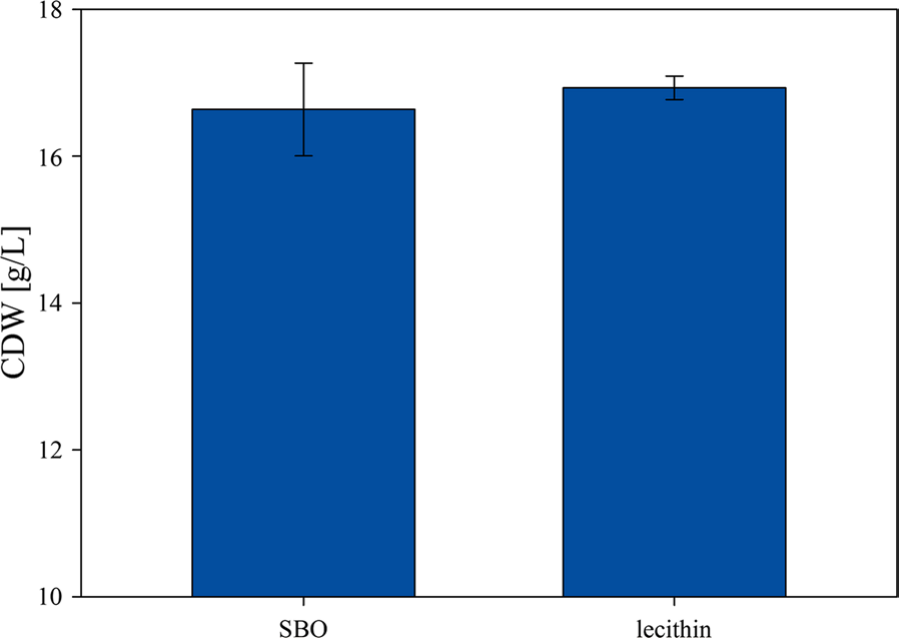
Six experiments were performed either with crude SBO or extracted lecithin as media supplement. The plot shows the variance between those experiments concerning biomass formation

Finally, we compared crude SBO and extracted lecithin in 6 reproducibility experiments. These results indicated less variations in biomass growth when extracted lecithin was used (4% variation for crude SBO and 1% for extracted lecithin; see standard variations in Fig. 6) arguing for an increased use of this less variable raw material compared to crude SBO.

In summary, we could identify lecithin as a kMA in crude SBO for the evaluated process. A fast and cheap extraction procedure for lecithin combined with an HPLC method for optimal dosage was developed facilitating the potential application of a less variable media supplement, namely extracted lecithin from crude SBO. The comparability of the effect of crude SBO and extracted lecithin was shown with respect to biomass formation. As growth and productivity are correlated in *P. chrysogenum* processes, we can assume an equal effect on productivity (Douma et al. 2010).

## Discussion

Complex raw materials, which are multicomponent mixtures of biological origin, are often used as cheap media supplements for different organisms. However, the specific kMA in these mixtures triggering the positive effects on biomass growth and productivity are mostly unknown. Furthermore, these raw materials underlie high lot-to-lot variability (Hofer and Herwig 2017; Hofer et al. 2018) which naturally leads to variations in the processes and contradicts QbD guidelines (Saha and Racine 2010) (ICH 2009). Vegetable oils, such as soy bean oil (SBO), belong to these complex raw materials and are often used as media supplements in fungal bioprocesses. However, current QbD guidelines demand for science-based knowledge of raw material quality and the knowledge of mechanistic correlations between raw material attributes and their effects. In this study we hypothesized that lecithin is the kMA in SBO triggering the positive effects on fungal growth and productivity. To test our hypothesis we adapted a rather simple extraction method for lecithin from SBO based on lecithin degumming and developed an analytical method allowing the supplementation of defined amounts of either crude SBO or extracted lecithin. In a series of supplementation experiments with a *P. chrysogenum* strain we could demonstrate that lecithin is in fact the kMA in crude SBO causing beneficial effects on fungal growth. We assume an equal positive effect on productivity due to the correlation between growth an productivity in this process (Douma et al. 2010). For both crude SBO an extracted lecithin, these effects were concentration-dependent. In reproducibility studies we found that extracted lecithin caused less variation in biomass growth than crude SBO, which would be highly favorable in production processes. As the method of lecithin degumming is already used in large scale refinery processes, we believe that it can also be introduced as raw material preparation step for bioprocesses. Alternatively, we propose to quantify the amount of lecithin in crude SBO to have a means of evaluation for this complex raw material and to be able to avoid over-dosage of the supplement and thus negative effects.

## Abbreviations

CAD: charged aerosol detection
CMA: critical material attribute
DoE: design of experiment
ESI–MS: electrospray ionization–mass spectrometry
FFA: free fatty acids
FAME: fatty acid methyl esters
FTIR: fourier transform infrared spectroscopy
HPLC: high performance liquid chromatography
ICP-OES: inductively coupled plasma-optical emission spectrometer
kMA: key material attribute
µ: growth rate
PCA: principal component analysis
PLS: partial least squares
QbD: quality by design
SBO: soy bean oil

## References

[CR1] Anderson RF, Tornqvist EGM, Peterson WH (1956). Effect of oil in pilot plant fermentations. J Agr Food Chem.

[CR2] Bateman HG, Jenkins TC (1997). Method for extraction and separation by solid phase extraction of neutral lipid, free fatty acids, and polar lipid from mixed microbial cultures. J Agr Food Chem.

[CR3] Bligh EG, Dyer WJ (1959). A rapid method of total lipid extraction and purification. Can J Biochem Phys.

[CR4] Carelli AA, Brevedan MIV, Crapiste GH (1997). Quantitative determination of phospholipids in sunflower oil. J Am Oil Chem Soc.

[CR5] Dijkstra AJ, Van Opstal M (1989). The total degumming process. J Am Oil Chem Soc.

[CR6] Douma RD, Verheijen PJ, de Laat WT, Heijnen JJ, van Gulik WM (2010). Dynamic gene expression regulation model for growth and penicillin production in *Penicillium chrysogenum*. Biotechnol Bioeng.

[CR7] Gao Y, Yuan YJ (2011). Comprehensive quality evaluation of corn steep liquor in 2-Keto-L-gulonic acid fermentation. J Agr Food Chem.

[CR8] Goldschmidt MC, Koffler H (1950). Effect of surface-active agents on penicillin yields. Ind Eng Chem.

[CR9] Hofer A, Herwig C (2017). Quantitative determination of nine water-soluble vitamins in the complex matrix of corn steep liquor for rawmaterial quality assessment. J Chem Technol Biot.

[CR10] Hofer A, Kamravamanesh D, Bona-Lovasz J, Limbeck A, Lendl B, Herwig C, Fricke J (2018). Prediction of filamentous process performance attributes by CSL quality assessment using mid-infrared spectroscopy and chemometrics. J Biotechnol.

[CR11] ICH (2009) Harmonised Tripartite Guideline: Pharmaceutical Development Q8 (R2)

[CR12] Jangle RD, Galge RV, Patil VV, Thorat BN (2013). Selective HPLC method development for soy phosphatidylcholine fatty acids and its mass spectrometry. Indian J Pharm Sci.

[CR13] Jones AM, Porter MA (1998). Vegetable oils in fermentation: beneficial effects of low-level supplementation. J Ind Microbiol Biot.

[CR14] Millis NF, Palmer BM, Trumpy BH (1963). Effect of lipids on citric acid production by an *Aspergillus Niger* mutant. J Gen Microbiol.

[CR15] Nash AM, Frankel EN (1986). Limited extraction of soybeans with hexane. J Am Oil Chem Soc.

[CR16] Ohta N, Park YS, Yahiro K, Okabe M (1995). Comparison of neomycin production from *Streptomyces*-*Fradiae* cultivation using soybean oil as the sole carbon source in an airlift bioreactor and a stirred-tank reactor. J Ferment Bioeng.

[CR17] Pan SC, Bonanno S, Wagman GH (1959). Efficient utilization of fatty oils as energy source in penicillin fermentation. Appl Microbiol.

[CR18] Posch AE, Spadiut O, Herwig C (2012). Switching industrial production processes from complex to defined media: method development and case study using the example of *Penicillium chrysogenum*. Microb Cell Fact.

[CR19] Posch AE, Koch C, Helmel M, Marchetti-Deschmann M, Macfelda K, Lendl B, Allmaier G, Herwig C (2013). Combining light microscopy, dielectric spectroscopy, MALDI intact cell mass spectrometry, FTIR spectromicroscopy and multivariate data mining for morphological and physiological bioprocess characterization of filamentous organisms. Fungal Genet Biol.

[CR20] Reese ET, Maguire A (1969). Surfactants as stimulantes of enzyme production by microorganisms. Appl Microbiol.

[CR21] Ruiz-Gutierrez V, Perez-Camino MC (2000). Update on solid-phase extraction for the analysis of lipid classes and related compounds. J Chromatogr A.

[CR22] Saha BC, Racine FM (2010). Effects of pH and corn steep liquor variability on mannitol production by *Lactobacillus intermedius* NRRL B-3693. Appl Microbiol Biot.

[CR23] Tarafdar JC, Claassen N (1988). Organic phosphorus compounds as a phosphorus source for higher plants through the activity of phosphatases produced by plant roots and microorganisms. Biol Fertility Soils.

[CR24] Wiedermann LH (1981). Degumming, refining and bleaching soybean oil. J Am Oil Chem Soc.

